# Systematic Review: Effectiveness of psychosocial interventions on wellbeing outcomes for adolescent or adult victim/survivors of recent rape or sexual assault

**DOI:** 10.1177/1359105320950799

**Published:** 2020-08-24

**Authors:** Jane Lomax, Jane Meyrick

**Affiliations:** 1 University of Bath, Bath, UK; 2 University of the West of England Bristol, Bristol, UK

**Keywords:** intervention, sexual health, sexual violence, systematic review, trauma

## Abstract

Sexual assault and rape are common forms of sexual violence/abuse. The psychological/health consequences represent significant and ongoing harm. It seems imperative that victim/survivors receive evidence-based support within first response settings. To assess what psychosocial interventions work for victim/survivors of a recent sexual assault. Twenty-seven electronic databases were systematically searched. Narrative data synthesis was used to read across studies. Reporting format follows PRISMA checklist. Ten studies were identifed including range of interventions. The evidence is sparse and scientifically weak, common flaws are reviewed. There is some weak evidence for the impact of video and cognitive behavioural therapy (CBT) based interventions, especially trauma processing. There is a gap in the evidence base on psychosocial interventions for victim/survivors of sexual assault and higher quality research is required.

This review aims to evaluate the effectiveness of psychosocial interventions for reducing the harmful impact of sexual assault and rape. These are common forms of sexual violence/abuse with 4.7% of women and 3.5% of men in the United States experiencing some form of contact sexual violence in the preceding twelve months. This includes 1.2% (1.5 million) women being raped (attempted or completed) and 0.7% men being made to penetrate someone (Smith et al., 2018). Factors such as shame, stigma and fear of reprisals result in under-reporting, thereby making exact prevalence difficult to assess (World Health Organisation; WHO, 2012).

The high prevalence of sexual assault is particularly concerning given the significant impact these experiences can have on physical and mental health (Dworkin et al., 2017; García-Moreno et al., 2013). Sexual assault carries the risk of physical injury (Kerr et al., 2003; Zilkens et al., 2017) and is associated with health consequences including sexually transmitted disease, pregnancy, sexual or gynaecological problems (Jina and Thomas, 2013) and somatic complaints such as pelvic pain (Clum et al., 2001). Worldwide the consequences of sexual assault and rape represent a significant public health burden (Jewkes et al., 2002).

In terms of mental health consequences, research shows that almost all female sexual assault survivors experience significant post-traumatic symptoms in the immediate aftermath of an assault, with around half continuing to experience these symptoms three months later (Feeny et al., 2004; Rothbaum et al., 1992). Interpersonal traumas such as sexual assault and rape are associated with higher rates of post-traumatic stress disorder compared to other types of trauma (Gilboa-Schectman and Foa, 2001; Kelley et al., 2009; Kessler et al., 2017). A WHO epidemiological study found that rape carried the highest conditional risk for PTSD (19.0%), nearly five times the aggregate risk after ‘any’ trauma type (4%; Liu et al., 2017). Indeed, a recent meta-analysis concluded that sexual assault was strongly associated with heightened risk for all forms of psychopathology (Dworkin et al., 2017).

Considerable research has focused on identifying effective psychological therapies for relieving this high burden of psychological suffering, especially the longer-term treatment of PTSD, depression or anxiety (Regehr et al., 2013; Vickerman and Margolin, 2009). The strongest evidence suggests that exposure based approaches such as cognitive processing therapy (CPT), prolonged exposure therapy (PE) and eye movement desensitisation and reprocessing (EMDR) have the greatest efficacy in treating symptoms of PTSD and depression, with improvements in anxiety, guilt and dissociation, depending on the treatment modality (Regehr et al., 2013; Vickerman and Margolin, 2009). These results are consistent with the finding that individual or group trauma-focused CBT (or exposure therapy), EMDR and stress management were effective in the treatment of PTSD in general trauma populations (Bisson and Andrew, 2007).

There have also been calls for effective early interventions for victim/survivors to alleviate immediate distress and prevent the development of longer-term problems (e.g. Campbell and Wasco, 2005). Understanding what works is especially important given that peri-traumatic distress is associated with increased risk for PTSD in all trauma survivors (Brewin and Holmes, 2003) and in sexual assault survivors specifically (Steenkamp et al., 2012). The early aftermath of sexual assault may be a critical period for determining survivor risk or resilience following a sexual assault (Dworkin and Schumacher, 2016).

Currently, support for victim/survivors is available from a network of rape crisis centres, originating in the feminist movement, and typically offering telephone or crisis support, advocacy and counselling (Robinson and Hudson, 2011). In the UK, Sexual Assault Referral Centres provide immediate help and support such as forensic medical exams, crisis support and counselling (NHS England, 2017). Sexual Assault Response Teams provide similar services in the U.S. (Greeson and Campbell, 2013). Reviews have identified how services should respond to disclosures of sexual assault in terms of immediate need (Lanthier et al., 2018), highlighting the importance of positive interactions in reducing symptoms of post-traumatic stress (Dworkin and Schumacher, 2016). However, there is a gap in the evidence around the role of wider psychosocial interventions for improving recovery. It is therefore critical that the evidence for early psychosocial interventions is reviewed.

Previous reviews have primarily focused on the effectiveness of interventions to *treat* the mental health consequences of sexual assault (e.g. Parcesepe et al., 2015; Regehr et al., 2013; Taylor and Harvey, 2009; Vickerman and Margolin, 2009). Most studies reported outcomes for survivors more than three-months post-assault, as symptoms may spontaneously remit or stabilise in this time (Rothbaum et al., 1992). Decker and Naugle (2009) provide a literature review of the evidence for crisis interventions for sexual assault survivors, however they concluded that research on early interventions after sexual assault is limited and did not assess scientific quality.

A previous systematic review has explored the role of early interventions in preventing the development of PTSD after sexual assault (Dworkin and Schumacher, 2016). However, this review included mixed trauma samples (physical/sexual assault) which, given the potential for differential or iatrogenic effects, limits the conclusions that can be drawn for victim/survivors of sexual trauma. Research has shown that trauma interventions may benefit some trauma populations but increase symptoms in others (Bisson et al., 1997; Mayou et al., 2000). This review focuses on all psychosocial interventions for sexual assault survivors only. Furthermore, prevention of PTSD is often not the only sole goal of such early interventions (Dworkin and Schumacher, 2016), therefore, this review broadens the scope to include all wellbeing outcomes with the hope this will capture a wider range of interventions, including current practice.

Thus, the objective of this review was to systematically identify and critically evaluate the quality of the evidence for the effectiveness of psychosocial interventions with adolescents or adults who have experienced a recent sexual assault or rape, encompassing all psychosocial wellbeing outcomes. More specifically, this review sought to: (1) examine the quality of studies that have investigated the effectiveness of early interventions for adolescent or adult survivors of a recent sexual assault and (2) summarise the effectiveness of these interventions, identifying gaps and common methodological flaws in order to inform evaluation practice in the field.

## Methods

The recommended standard for reporting systematic reviews, the PRISMA checklist (Moher et al., 2009) guided this review. Studies were included in this review based on the following inclusion criteria:

### Eligibility criteria

Studies that evaluated the effectiveness of an intervention on survivors (⩾15 years old) of a recent sexual assault (⩽3 months). Adolescents are at high risk of sexual assault (ONS, 2018) and 15 years was set as the lower age limit following the WHO reports (García-Moreno et al., 2005; 2013).

- Studies that evaluated a psychosocial intervention, in any environment, by any provider, over any time frame and with any, or no, comparison group.
- Original studies published prior to 6th April 2019 available in English. There was no research budget to accommodate translation.
- Studies that had a quantitative evaluation of effect with at least one measure of any psychosocial wellbeing outcome pre- and post-intervention excluding case reports (<4 cases; Abu-Zidan et al., 2012) given their limited generalisability and utility for establishing causal relationship (Nissen and Wynn, 2014). Studies that evaluated interventions to prevent or reduce sexual assault risk were excluded.

## Information sources

Systematic literature searches were conducted up to 6th April 2019 using 27 databases including Cinahl, Cochrane Collaboration, Emerald, Medline, PsychARTICLES, PsychINFO, SAGE Journals online, ScienceDirect and Taylor and Francis. Sources of grey literature were included to reduce the risk of publication bias (Rosenthal, 1979) and two UK experts were contacted by email to request additional published or unpublished studies they might be aware of. Neither approach yielded suitable studies. Relevant journals were hand searched, selected as likely publishing outlets for sexual violence research (Jordan, 2009). The reference lists of all included articles and relevant previous reviews were examined. See Appendix 1 for full details.

### Search strategy

Databases were searched using combinations of population (e.g. Sex* Assault, Rape, Sex* Violence) and intervention search terms (e.g. Psycho*, Suppor*, group, intervention, prog*, social, service* etc) connected with Boolean operators (see Appendix 1).

### Study selection

**Figure 1.**
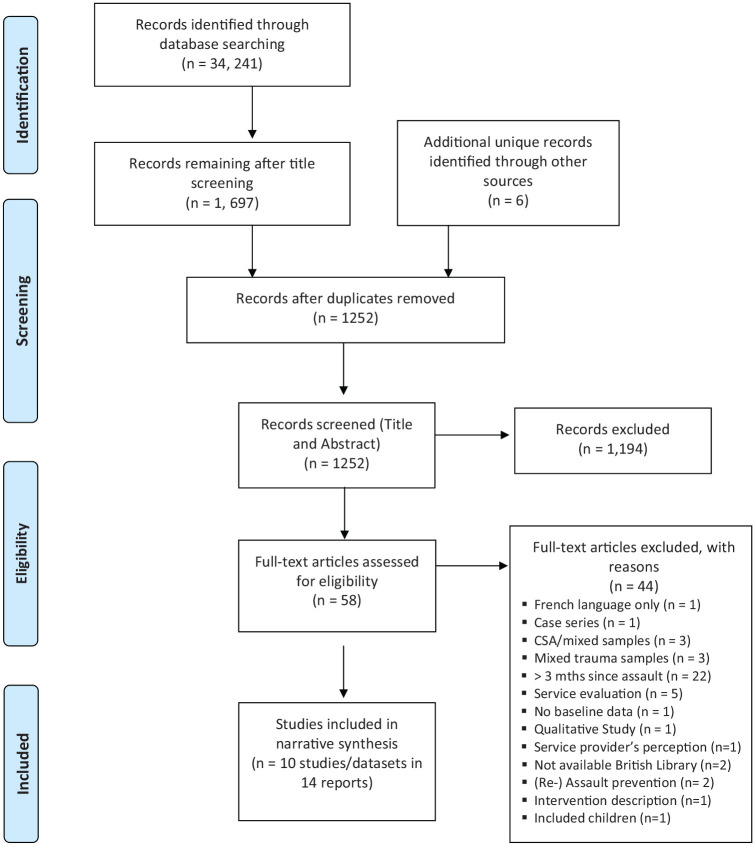
PRISMA 2009 flow diagram.

Titles and abstracts were systematically screened to identify potentially relevant studies. Two independent reviewers screened full copies of articles to identify relevant studies based on inclusion and exclusion criteria (see Figure 1 for results). Disagreements were resolved by discussion.

### Data collection process and data items

Two independent reviewers extracted data from included articles using a data extraction form. An initial trial confirmed relevant areas (see Appendix 1), ensured criteria were applied consistently and that consensus could be reached in case of discrepancy. Where examination indicated that articles were based on the same datasets, studies were considered together and are shown together in the data extraction table (see Table 1).

**Table 1.** Summary of study characteristics and data extraction table.

*Effectiveness of video interventions at time of forensic medical exam*								
Study and aim	Setting	Participants	Sample size and characteristics	Design	Intervention	Outcomes measured and time points assessed	Success of follow up	Key results within and between groups
**Miller et al. (2015)** Assess efficacy of video to reduce distress after a forensic medical post-sexual assault exam	SANE programme at USA hospital	• Women • 18 yrs • English speaking • Attending for forensic medical (SANE) exam within 72 hrs of sexual assault • 179 eligible	• **N** = **164 (91.6%)** • **Mean Age** = 28.79 yr, SD = 10.47, range 18-70 yrs. • **Ethnicity:** 61.5% White; 15.5% Black; 23% other • **72% previous sexual assault** • **Index assault:** 57% completed rape • More completed rape in standard care (67.1%: 46.8%).	**Controlled Clinical Trial** **Video (n** = **94)** *versus* **Standard Care (n=85)** RA, IBA	**Psychoeducational Video (VI; 9 mins)** • Psychoeducation on reactions to sexual assault, methods for graded exposure and targeting avoidance, strategies to improve mood **Standard Care (SC)** • Rape crisis advocate provided info on exam and services.	• Subjective Units of Distress (SUDS) • The PTSD-Symptom Scale Self-Report (PSS-SR) • State-Trait Anxiety Inventory (STAI) – State component only • **Pre** • **Post** • **2 weeks** • **2** month**s** • Follow up data collected by phone.	• n = 164 tx completion • n = 69 at 2 weeks • n = 74 at 2 months	• **VI** versus **SC on STAI scores at 2 week** [F(1, 68) = 6.82, p < .05, partial h2 = 0.094; mean difference = 8.60, SEdiff = 3.41] • **and 2m FU** [F(1, 74) = 4.58, p < .05, partial h2 = 0.06; mean diff = 6.66, SEdiff = 3.11] • N.s. for VI versus SC on PSS-SR / SUDS • **At 2week:** VI (no sexual assault history) had lower total severity score on PSS-SR (mean diff = -12.61, p=.011). N.s. at 2m.
**Resnick et al. (2007a)** *includes Acierno et al. (2003) - Examine efficacy of a two-part video in reducing post-sexual assault substance use and abuse.	A Major University Hospital South East USA	• Women • ⩾ 15 yrs old • Attending for SANE (sexual assault nurse examiner) exam within 72 hrs of sexual assault • 592 eligible	• **N** = **442 (74.7%)** • **Mean Age:** NV (n=107) Mean = 26.49 years (SD=10.4); AV (n=161) mean 25.93 years (SD = 10.24). • **Majority Single (**81.6%) • **Ethnicity:** 58.2% White; 38% Black; 3.8% other. • 59.3% Lifetime sexual or physical assault • **Index assault:** 92.5% included penetration.	**Controlled Clinical Trial** Any Video (n=283) *versus* Standard Care/Non-Video (n=159) RA, IBA	**Any Video (AV)** • Full Video (17 mins) • Medical Exam Prep Video (7mins 40secs; Info about medical exam with model demonstrating coping). • Psycho-Education (10mins; see above plus brief strategies to target substance misuse) • Standard Care/Non-Video • Rape crisis counsellor attends exam.	• Alcohol and Substance Misuse (Lifetime/Pre-assault potentially problematic use) and abuse (DSM-IV) via clinical interview • ƒ of use during previous 2 weeks – self-report at follow up • **Baseline** • **T1** – < 3m (M=48.94 days, SD=11.14) • **T2** – 3-6m (M=104.83 days, SD=19.55) • **T3** – 6m or more (M=196.37 days, SD = 79.27).	• 406 tx completers • AV; n= 247 (87%) • 268 (66%) completed one FU. NV; n = 107 AV; n = 161	• Reduced ƒ marijuana use in AV among pre-sexual assault marijuana users at: • **T1**: F (7206) = 19.39, p < .001; • **T2**: F (7122) = 12.28, p < .001; • **T3**: F (7206) = 14.48, p < .001; • No effect of AV on alcohol or hard drug use/abuse at FU after controlling for other predictor variables.
**Resnick et al. (2007b)** * Includes Resnick et al. (2005) and Resnick et al. (1999) -Evaluate efficacy of video prior to forensic medical exam to reduce mental health symptoms at FU.	A major South Eastern USA university hospital	• Women • ⩾ 15 yrs old • Attending for SANE exam within 72 hrs of sexual assault • 268 eligible (rape or suspected rape)	• **n** = **225 (84%)** • **Mean age** = 26.1 yrs (SD = 9.8) • **Majority single (90%)** • **Ethnicity:** 48% African American, 50% White, 2% Other • **n** = **46 (37%) reported previous history of rape.** • **100% rape or suspected rape** • No baseline diffs apart from VI group had higher baseline distress (controlled in analysis).	**Controlled Clinical Trial** Video (n = 117) *versus* Standard Care (n = 108) RA, TAM, IBA	**Video Intervention** (17 mins) • Medical Exam prep video with info about the exam with model coping. • Psychoeducation video on psychological reactions to sexual assault, method for graded exposure and targeting avoidance, strategies to improve mood	• Subjective Units of Distress (SUDs) • The PTSD Symptom Scale – Self report (PSS-SR) • Beck Depression Inventory (BDI) • Beck Anxiety Inventory (BAI) • Family Resource Scale (FRS) • **PRE** • **POST** • **T1**: 6 week (mean = 58.64 days, SD = 22.7) • **T2**: 6m (mean = 184.75 days, SD = 50.97). • FU interview	• n = 205 TX completion (83% video) • completed one follow up n = 140 (68.3%) • T1: n = 123 (60%; 61-Vid; 62-SC) • T2: n = 128 (68.3%; 62 video, 66 non-video)	**For women with prior rape history:** • **T1**: **Video** versus **SC had lower PSS-SR** (CR = -3.45; 90% CI for *B*: -18.95 to – 2.75; r = -0.28; medium ES). N.s at T2. • **T1: Video** versus **SC had lower BDI** (CR = -2.88; 90% CI for *B* = -18.89 to -1.04; r = -0.24; small-medium ES) and **T2** with smaller ES (CR = - 1.54; 90% CI for *B*: -14.40 to -3.61; r = -0.13) **For those with no prior rape history:** • **At T1 Video associated with increase in PSS-SR** (CR = 1.32; 90% CI for *B*: -3.50 – 10.87; r = 0.11; small effect size) **and higher BAI** (r = 0.15; CR = 1.71; 90% CI for *B*: -3.03 to 14.89; r=0.15; modest effect size). N.s at T2. • N.s. effect on BDI at T1/T2
**Walsh et al. (2017)**. Assess efficacy of video to reduce drug and alcohol use after a sexual assault.	One of two medical centres in a Mid westerncity area (USA)	• Women • ⩾15 years old • English speaking • 711 eligible (466 excluded)	• **N** = **245 (34.5%)** • **Mean age** = 27.5 yrs (SD = 9.3) • Married/cohabiting 13.6%; Single 74%; Divorce/widow 12.3% • Minority ethnic status 57.1% • Student 13.6%; Employed 34.4% • Prior sexual assault 61.7% • Past year binge drinking 44.2% • Past-year marijuana use 46.8%	**Randomised Controlled Trial** **Prevention of Post-Rape Stress Video** (n= 77) *versus* **Pleasant Imagery and Relaxation Video** (n = 77) *versus* **Treatment as usual** (n = 79) RA, IBA	**Prevention of Post-Rape Stress Video (PPRS; 9 mins)** • Medical exam preparation. • Psychoeducation on sexual assault, methods for targeting avoidance and graded exposure, alternative non-substance coping strategies. **Pleasant imagery and Relaxation Video (PIRI; 9 minutes)** • Diaphragmatic breathing, muscle relaxation, pleasant imagery and sounds. **Treatment as Usual** Completion of SANE exam	• **Alcohol and Marijuana Use:** ƒ of use, past 2weeks at FUs. Self-report alcohol use (days x drinks) or marijuana use (days) • **Problematic Alcohol and Drug Use:** Alcohol Use Disorder Identification Test (AUDIT) and Drug Abuse Screening Test (DAST) to assess in year prior to rape at T1; since rape at T3 • **Baseline** • **T1: 2** month**s** (M days = 56.95, SD = 24.87) • **T2: 3.5** month**s** (M days = 107.63, SD = 25.17) **T3: 6.5** month**s** (M days = 195.20; SD = 55.38).	• **N** = **233 tx completers** • **T1:** 66% (n=154) • **T2:** 88% of these (n = 135) **T3:** 79% of these (n = 121)	• N.s. main effect of VI. **In past** year **binge drinkers:** • T3: PPRS versus TAU lower log odds of alcohol use (p < .0004). N.s. trend for PPRS versus PIRI. **Minority Status:** • T3: PPRS versus TAU n.s. trend for lower alcohol use in minority women and lower DAST in non-minority. **Marijuana Use:** • For those with *no* past year marijuana use: **T1**: PPRS versus PIRI = lower use (p < .0004) and **T3:** PPRS v TAU = lower use (p < .0004). • **T1 and T2:** Those *without* prior sexual assault history: PPRS versus PIRI fewer days marijuana use (p < .0004). • **T3:** Those *with* prior sexual assault history: PPRS versus PIRI less marijuana use (*p* < .0004).
*Effectiveness of Individual Cognitive-Behavioural Based Interventions*								
**Anderson and Frank (1991)** * Includes Frank et al. (1988) To compare outcomes for 4 txs: Cognitive behavioural therapy (CBT); Systematic Desensitisation (SD) Psycho-educational Intervention (PEI); Psychological Support (PS).	Referred by two rape crisis centres in Allegheny County, Pittsburgh, USA	• Women • < 1 month post sexual assault • 532 eligible;	• **Total n** = **231 (51.3%)** • CBT-SD: n =60; mean age 23.3yrs (SD = 7.4) • PEI-PS: n=88; mean age is 25.4yrs (SD=9.0) • Majority single (86%) • Ethnicity: CBT and SD: 81.4% Caucasian; 18.6% African American; PEI and PS: 69% to 31% African American. • 100% rape • No baseline diffs between groups.	**Controlled Clinical Trial** CBT (n = 50) vs SD (n = 49) versus PEI (n = 69) versus PS (n = 63) RA, TAM for CBT-SD; Session content specified	• **CBT (14hrs)** reduce avoidance address unhelpful thoughts • **SD (14hrs)** Progressive muscle relaxation; imaginal exposure. • **PEI (4hrs)** Info on rape reactions, how to manage them, rape myths and reactions of others, support • **PS (4hrs)** Control for benefit of support alone. • **Therapists** were clinical psychs or psychiatric s/w • **Weekly Individual sessions**	• Beck Depression Inventory • Modified Veronen-Kilpatrick Fear Survey • Depressed versus not depressed ( < 16 on BDI) • **PRE** • **POST** **3** , **6** , **12** month**s** FU	Tx Completion: • CBT: n = 34 (68%) • SD: n = 26 (53.1%) • PEI: n = 48 (69.9%) • PS: n = 40 (63.5%)	• CBT and SD and PEI and PS significant decrease on *depression* and *fear* (p < 0.0001) across time points. • N.s. difference between CBT v SD or PEI v PS, or CBT-SD versus PEI-PS at any time point. • **Clinically signif. depression**: At 3m 10% of CBT-SD versus 28% of PEI-PS, p < 0.01.; Trend at 6 mth, p =0.09; n.s. at 12m (21.1% versus 10%).
**Echeburúa et al. (1996)** Compare cognitive restructuring and coping-skills training (CR/CS) progressive muscle relaxation training (PR) in tx of acute stress disorder in victims of sexual aggression (over 1 year time).	Psychological counselling centre for women, Basque country, Spain	• Female • ⩾ 15 yrs old • 4-13 weeks post-assault (mean = 5) • Psychological treatment seekers • Meet criteria for Acute PTSD (DSM-III-R) • Screened 31	• **N** = **20 (66.6%)** • **Mean age** = 22 yrs (SD = 6.9); range = 15-45 yrs • 85% Single; 15% married. • **Ethnicity**: n/r • **100% rape or attempted rape** • No baseline imbalances	**Controlled Clinical Trial** (two group design with repeated measures) N = 10 in each group RA	• **Cognitive Restructuring and Coping Skills Training (CR/CS)** Psycho-education, cognitive model, thought modification/stopping; progressive muscle relaxation, cognitive distractions and gradual exposure techniques. **Progressive Muscle Relaxation training (PR)** • Both individual and weekly • Clinical psychologist • 5 hrs CR/CS; 4.15hr PR	• *Clinical Interview Scale of Severity of PTSD Symptoms* (DSM-III-R). • *Diagnosis of PTSD* • *Beck Depression Inventory* • *State-Trait Anxiety Inventory* • *Modified Veronen and Kilpatrick Survey of Fears* • *Scale of Adaptation* • Assessed during therapist i/v • **Pre Tx; Post Tx** • **1, 3, 6, 12 month FUs**	No Dropouts	• CR/CS lower *PTSD symptoms* than PR; trend from post (p < .1) but by 12m FU (Mean = 5 (SD = 2.49); versus Mean = 10.5 (SD – 7.16), t = 2.30, p < .05). • Most evident in *re-experiencing and avoidance* subscales. • All other between group outcomes n.s (fears, anxiety, depression or inadapation). • *% PTSD diagnosis*: n.s. at any time but at POST: 20% of CR/CS and 50% of PR. At 12m 0% of CR/CS and 20% of PR
Kilpatrick and Veronen (1984) **Paper not available from British Library. Info gained from references e.g. Foa et al, (1993), Anderson and Frank (1991) and Vickerman and Margolin (2009).	Not reported	Rape survivors 6-21 days post-assault Recruited from rape crisis centre (adults)	• **N=15** • All women • Ethnicity/Age other details not reported. • Victims randomly allocated to one of three conditions.	**Controlled Clinical Trial** BBIP (n= 10) versus Repeat Assessment (pre, post, 1, 2 and 3m). versus Delay Assessment (pre, post and 3m)	• **Brief Behavioural intervention Procedure (BBIP).** Re-experiencing event, express feelings, psycho-ed on fear cycle, guilt/blame, coping skills. • 4-6 hours contact (2 sessions) • Standardised tx delivered by peer counsellors.	• Veronen and Kilpatrick Modified Survey of Fears • Sexual dysfunction • Depression • Anxiety • **Pre** • **Post (6-21** day**s)** • **1, 2 & 3 month FUs**	• Dropouts n/reported	• No significant diffs between BBIP, RA, DA conditions • All participants reported reductions on measures of psychopathology at the 3-month assessment (within groups).
**Nixon et al. (2016)** Examine effect of brief cognitive processing therapy (B-CPT) compared with active treatment as usual (TAU) for survivors of recent sexual assault with Acute Stress Disorder (ASD) Assessment over 1-year period.	A community sexual assault centre in Adelaide Australia	• Consecutive clients seeking tx at sexual assault crisis centre • > 18 yr old • Rape/sexual assault in past month • **Met criteria for ASD** • If applicable - stable on meds for 4 weeks. • N = 57/158 eligible.	• **N** = **47 (82%)** • **B-CPT:** 1 male; 23 female (Mean age = 32.46 yrs, SD = 11.43) • **TAU:** 22 female (Mean age = 29.95 yrs, SD = 8.48) • **Mostly Caucasian** • 86% had co-morbid diagnosis, 77% previous sexual trauma, 30% psych admission. • Clinically negligible diffs on baseline variables	**Controlled Clinical Trial** Brief-CPT (n=25) *versus* TAU (n = 22) Sequential RA, TAM, IBA, ITT	• **Brief Cognitive Processing Therapy (B-CPT)** Modified CPT protocol; cognitive restructuring, writing and processing trauma. • 6 weekly sessions (90mins) • **Treatment as Usual (TAU).** Eclectic community practices. Not systematic CBT/exposure • Average 3.5 sessions • TAU received 4-5 extra sessions post tx phase. • 9 female therapists RA to CPT or TAU.	• Clinician administered PTSD scale (CAPS) • PTSD Checklist Self-report (PCL-S) • Post-traumatic Cognitions inventory (PTCI) Beck Depression Inventory (BDI) • **Pre** • **Post (1 week tx)** • **3, 6 & 12 months FU**	• **N** = **46 tx completed** • **B- CPT** • POST n = 15 • 3m n =11 • 6m n = 10 • 12m n = 12 **TAU** • POST n = 17 • 3m n =13 • 6m n = 14 • 12m n = 13	• Both B-CPT and TAU groups demonstrated large and clinically significant reductions in PTSD (ES: 0.76 – 1.45) and depression (ES: 0.42- 0.92). Moderate - large ES for PTCI reduction (0.42 to 0.94) at each FU • Smaller **between group** effect sizes typically favoured CPT (ES: 0.13 – 0.50 posttraumatic stress and 0.13-0.41 depression) over the course of FUs • N.s. diffs in PTSD diagnosis • Independent assessment of PTSD severity: more CPT group reached good end state functioning at 12m (50%) versus TAU (31%), p=0.32 • Comparable Adverse effects
**Rothbaum et al. (2012)** To examine effect of modified prolonged exposure therapy on posttraumatic stress reactions at 4 and 12-weeks post-trauma.	Public Hospital Emergency department, largest in Georgia, USA	• Mixed trauma sample: rape subgroup • **Age** 18 – 65 yrs • within 72 hrs. • Met Criterion A of DSM-IV for type of trauma. • Acute stress higher in intervention (controlled in analysis).	• **Mixed trauma N** = **137** • **N** = **47 for rape trauma subgroup** • Age/Ethnicity other demographics not reported for subgroup. • **100% rape**	**Randomised Controlled Trial** **Modified Prolonged Exposure** (n = 28) versus **Assessment only** (n = 19) RA, TAM, IBA	**Modified Prolonged Exposure** • Imaginal and in vivo exposures to trauma memories or rape related cues. Psycho-education on trauma, breathing training, homework. • 3 sessions, weekly (1hr long) • Therapists trained in PE and modified protocol to MSc/Doctoral level.	• Standardised Trauma Interview • PTSD Diagnostic Scale (PDS) • Immediate Stress Reaction Checklist (ISRC) • PTSD Symptom Scale – clinician administered (PSS-I) • Beck Depression Inventory (BDI) • **Pre; 4-week FU; & 12-week FU**	• n = 102 (74%) at 4week FU. • n = 91 (66%) at 12week FU. • Subgroup figures n.r. • Majority in person. Some by phone/mail	**For rape subgroup:** • At 4 week: PE group had significantly lower PSS-I scores (M=20.10, SE = 2.38) versus assessment (M =30.45, SE = 2.73), with large ES (Cohen’s d =0.7, p < .01) • At 12 week: (M = 16.63, SE= 3.05) versus assessment (M = 25.04, SE = 3.37) with large ES (Cohen’s d = 0.52; p=.05). Results for rape subgroup and Depression not reported. Nor previous trauma PTSD (PDS)
**Tarquinio et al. (2012)** - To test effectiveness of early EMDR on the psychological consequences of rape.	Referrals to French research centre (n=6); from GP (n=5); or regional support agencies (n= 6).	• First sexual assault experience • Between 24 – 72hr post-assault • Age 18-60yrs	• **N** = **17** • **All female** • **Mean age** = **32.2 yrs (SD** = **9.1)** • Cohabiting 53%; Married 23.5%; Single 23.5% • **100% rape**	Cohort – one group repeated measures.	**URG-EMDR (1 session)** • Imaginal exposure to trauma, emotional pts identified, desensitisation with rapid eye movement until SUD of 2-3 reached. • Average duration is 1h 53 mins (SD = 48.7 mins) • 1 to 2 h (13/17); 2 to 3 h (3/17); 3 h /more (1/17) • Psychologists	• The Intrusion of Events Scale (IES) • Self-Report Sexual Function • Subjective Units of Distress (SUD) • **Pre** • **Post** • **4-week** • **6-months**	No dropouts reported.	• Improvement between pre-post on **IES** [total Wilks’s λ score (3, 14) = .09, P < .001; Wilks’s λ score for intrusion (3, 14) = .07, P < .001 and Wilks’s λscore for avoidance (3, 14) = .18, p < .001] and **SUD** (Wilks’s λ (3, 14) = 069, p < .001). N.s at other FUs. • Levels of desire (Wilks’s λ (3,14) =.12) and excitation (Wilks’s λ (3,14) =.09) improve at 4weeks (p < .0001) then stabilised.

*Note*: n.r.; not reported; n.s.; not significant; yr; year; m; month; tx; treatment; RA; random assignment; TAM; treatment adherence monitored; IBA; independent blind assessor, s/w; social worker, POST; post-treatment; FU; follow up assessment; ITT; intention to treat analysis, ES; Effect Size. Significant values have been presented in bold.

### Scientific quality - risk of bias

Studies were evaluated on scientific quality or to what degree the results could be attributed to the intervention through steps such as blinding, randomisation, sample size etc. Within this field, higher strength studies such as double-blind randomised control trials are unlikely, however, but steps towards rigour were assessed at study level using the standardised Cochrane risk of bias tool (Higgins and Green, 2011) at study level, by two unblinded independent reviewers. Scientific quality was used in the narrative synthesis to frame the validity of study effect findings. This is summarised in Table 2 in Appendix 1. Common methodological flaws or risk of bias across studies was also summarised in the narrative synthesis.

**Table 2.** Appraisal of the risk of bias of the included studies using the Cochrane risk-of-bias tool.

Authors	Random Sequence Generation	Allocation Concealment	Blinding of participants and personnel	Blinding of outcome assessment	Incomplete outcome data	Selective Reporting	Other Bias	Overall
Anderson and Frank (1991)	**Unclear**	**Unclear**	**Low**	**Unclear**	**Unclear**	**Unclear**	**High**	**Unclear**
Echeburúa et al. (1996)	**High**	**High**	**Low**	**High**	**Low**	**Low**	**Low**	**High**
Kilpatrick and Veronen (1984)	**Unclear**	**Unclear**	**High**	**Unclear**	**Unclear**	**Unclear**	**Unclear**	**Unclear**
Miller et al. (2015)	**Unclear**	**Unclear**	**High**	**Low**	**Low**	**Low**	**High**	**Unclear**
Nixon et al. (2016)	**High**	**Low**	**Low**	**Low**	**Low**	**Low**	**Low**	**Low**
Resnick et al. (2007a)	**Unclear**	**Unclear**	**High**	**Low**	**Low**	**Low**	**Low**	**Unclear**
Resnick et al. (2007b)	**High**	**High**	**High**	**Low**	**Low**	**Low**	**Low**	**High**
Rothbaum et al. (2012)	**Low**	**Low**	**High**	**Low**	**Low**	**Low**	**Low**	**Low**
Tarquinio et al. (2012)	**High**	**High**	**High**	**High**	**Unclear**	**Low**	**High**	**High**
Walsh et al. (2017)	**High**	**Low**	**Low**	**Low**	**Low**	**Low**	**Low**	**Low**

### Summary measures

Measures extracted were differences between reported mean scores for the intervention and control/comparison groups, pre- and post-intervention or within-groups where there was no control/comparison group. For studies which included a control or comparison group, the significance level of results (*p*-values) and effect sizes are included (where reported).

### Synthesis of results

Included studies varied in design, interventions evaluated, and in primary and secondary outcome measures. Therefore, meta-analysis of data was not appropriate (Liberati et al., 2009) and findings were synthesised narratively.

## Results

The systematic identification of studies resulted in 14 reports of 10 studies or datasets. Reasons for exclusion were documented using a PRISMA flow chart (see Figure 1). An overview of studies will be presented followed by effectiveness findings structured by type of intervention.

### Study characteristics

Four studies recruited participants presenting for a forensic medical exam (USA) within 72 hours or 7 days post-assault. One study recruited from a hospital emergency department (USA) within 72 hours; another via referral to a French research centre 24–72 hours post-assault; three from community rape crisis centres (USA, Australia) less than one-month or 6–21 days post-assault; one from a women’s counselling centre (Spain) 4–13 weeks post-assault.

### Sample size and characteristics

Studies included *N* = 1543 participants, with sample sizes from 17– 442; mean = 143.8. All but one participant was female. The age range was 15–70 years, mean = 24.9 years, excluding two studies where mean age was not reported (*N* = 62 adults).

Where reported, 50–87% of the sample were White Caucasian with 15.4– 48% African American or 2–23% other minority ethnic groups. One study grouped ethnicity as 57.1% minority ethnic and four did not report details. Most participants (90%) had experienced rape, attempted rape or suspected rape, the remaining reporting ‘rape or sexual assault’. Five studies reported on previous sexual assault history (58% of women had experienced this). Taken together this provides some evidence that the review findings may be relevant to rape crisis settings, working with women from a range of demographic groups and with some complexity in terms of trauma history.

### Study design

All studies used experimental repeated measures designs, with two randomised controlled trials, seven controlled clinical trials and one single cohort design.

### Interventions

Ten studies evaluated thirteen interventions seeking to improve psychological well-being by reducing psychological distress or substance misuse associated with the sexual assault.

### Content

Four studies evaluated an educational video (medical exam prep and/or psycho-education) either in full or as a component (Miller et al., 2015; Renick et al., 2007b; Resnick et al., 2007a; Walsh et al., 2017). The remaining six studies evaluated CBT based interventions including systematic desensitisation (SD); CBT (Anderson and Frank, 1991), cognitive restructuring and coping skills training (CR/CS; Echeburúa et al., 1996), Brief Behavioural Intervention (BBIP, Kilpatrick and Veronen, 1984), Brief Cognitive Processing Therapy (B-CPT; Nixon et al., 2016), Modified Prolonged Exposure (PE; Rothbaum et al., 2012) and finally single session Eye Movement Desensitization and Reprocessing (EMDR) (Tarquinio et al., 2012).

### Delivery

Video interventions were provided by nurses in the emergency department in four studies; in five studies interventions were delivered by mental health professionals, with details unclear in one. Interventions provided individually ranged from one session to 14 weeks, total treatment time from 7 minutes 40 seconds to 14 hours.

### Controls

In six studies, standard care (forensic medical exam), delayed assessment or repeated assessment only conditions were used as controls. Six studies used active controls; a pleasant imagery and relaxation instruction video, a psychoeducational intervention, psychological support, progressive muscle relaxation and standard clinician practice within a rape crisis counselling centre. One study lacked any comparison or control.

### Outcomes and measures

The primary psychological outcomes assessed included post-traumatic stress, depression, anxiety, and distress-fear. All studies used multiple previously validated measures or clinical interview to assess mental health symptoms or diagnoses (see Table 1 for details). Other outcomes were substance misuse or sexual dysfunction. All studies employed pre- and post-intervention measures, with follow up from 2 months to 1 year.

Six studies assessed post-traumatic stress symptoms and/or PTSD symptom severity using clinical interview or self-report measures based on the DSM-IV criteria for PTSD. Five studies assessed depression symptoms using the Beck Depression Inventory (BDI), either self-report or in an assessment interview. Three studies assessed anxiety using the State-Trait Anxiety Inventory (STAI) or the Beck Anxiety Inventory (BAI).

Three studies used the Modified Veronen-Kilpatrick Fear Survey to assess rape-related fears. Two studies examined self-reported sexual function. One study examined substance misuse via clinical interview or using Time-Line Follow-back. A further study assessed problematic alcohol and substance misuse using the 10-item Alcohol Use Disorder Identification Test (AUDIT) and the Drug Abuse Screening Test (DAST) respectively. Participants also assessed current alcohol and marijuana use based on self-reported use over the previous 14 days.

### Scientific Quality - Risk of Bias within studies

Table 2 in Appendix 1 presents the risk of bias for each study, assessed using the Cochrane Risk of Bias tool. Each study was assessed for the risk of bias in seven key areas and given an overall rating (Higgins and Green, 2011). The authors considered the internal validity of each study before considering external validity and the precision of the results.

### Summary across studies

Overall, studies evaluating psychosocial interventions were of poor methodological quality. Studies with poor internal validity lacked a comparison, a wait list control or treatment intensity varied between comparator conditions. Most studies did not use or report appropriate random allocation or blinding methods (which is challenging and may not be appropriate in this field). Random error may have been introduced through small sample sizes, the use of measures with questionable validity, the failure to record treatment fidelity or adherence in intervention or control conditions, and ‘treatment as usual’ being insufficiently described. External validity was often limited by insufficient attention to selection biases, participant attrition, and the lack of a control for spontaneous remission. These methodological flaws, and the heterogeneity of interventions and outcomes, point to a need for more rigorous research in this field, including standardising outcome measurement and approaches to CBT therapy delivery that would permit verification and comparison. Notably, the studies span a broad time frame (1984–2017) with methodological quality, reporting standards and potentially the quality of care and interventions likely to have changed (hopefully improving) over time.

### Effectiveness of studies

Results of individual studies evaluating the effectiveness of psychosocial interventions are presented in Table 1. They are grouped into two intervention types, psycho-educational videos and CBT-informed interventions in a narrative synthesis.

#### Effectiveness of video interventions at time of forensic medical exam

Four studies evaluated a brief CBT based video intervention provided at the time of a forensic medical exam within 72 hours to 7 days after a rape or sexual assault (Miller et al., 2015; Resnick et al., 2007a; 2007b; Walsh et al., 2017) with mixed results.

Resnick et al (2007a) used a two-component video with women ⩾15 years, attending hospital for a forensic medical exam within 72 hours post-assault. Video components were *Medical Exam Prep* and *Psychoeducation*, including instruction in self-directed exposure for trauma and strategies to reduce behavioural avoidance and substance use. Small sample sizes at follow-up precluded a dismantling study, therefore, the authors compared *Any Video* with *No Video* (forensic exam accompanied by a rape crisis counsellor). *Any video* was associated with reduced frequency of marijuana use for those using marijuana in the previous six weeks at all follow-ups across a six-month period (T1: F (7206) = 19.39; T2: F (7122) = 12.28; T3: F (7206) = 14.48, all p < .001). They found no association of the video with alcohol or hard drug use or abuse at follow up, after controlling for other predictor variables (e.g. history of sexual assault, pre-assault use). The risk of bias was unclear, with some details not reported. High levels of attrition may have enhanced the effect, although some participants only watched *Medical Exam Prep* which may have offset this.

Walsh et al. (2017) recruited women ⩾15 years, attending hospital for a forensic medical exam within 7 days post-assault. They examined the effect of a psychoeducational video (*PPRS)* on drug and alcohol consumption over a 6-month period, compared to an active control, *Pleasant Imagery and Relaxation Instruction (PIRI)* video and treatment as usual (TAU; specialist forensic examination). In women reporting past year binge drinking, those in PPRS had lower alcohol use at six-month follow-up versus TAU (β = -0.90, SE = -0.96 (0.25), *R*^2^*=0.21, p* < .0004), with a non-significant trend observed against PIRI and in minority women for PPRS versus PIRI. Among those reporting no marijuana use in the past year, those in PPRS reported less marijuana use at two-months versus PIRI (β = 0.84, SE =7.14 (0.67), *R*^2^*=0.22, p* < .0004) and at 6-months versus TAU (β = 0.67, SE = 9.74 (1.12), *R*^2^*=0.29, p* < .0004). Among those *without* a prior sexual assault history PPRS was associated with fewer days marijuana use versus PIRI at two-months (β = 0.24, SE = 3.21(0.86), *R*^2^*=0.22, p* < .0004) with a trend at three-months. However, at 6-months, in women *with* a prior sexual assault history, those in PPRS reported fewer days of marijuana use versus PIRI (β = 0.02, SE = 0.23 (0.92), *R*^2^*=0.29*, p < .0004). There were no significant effects on measures of problematic alcohol or hard drug use. The PPRS intervention may be effective at reducing substance use for some recent sexual assault victims over a six-month period, however the authors point to the complexity of the interaction between an individual’s experience of trauma, re-victimisation, substance misuse and the lack of impact on more problematic use. Overall, this study was at low risk of bias, however some participants received the intervention prior to the exam (*n* = 28; 18%) and some post (*n* = 126; 82%), which may have impacted results.

Resnick et al (2007b) used the full video with women ⩾15 years, attending hospital for a forensic medical exam within 72 hours of assault. At 6weeks post-assault, women *with* a prior sexual assault history, viewing the video *before* the forensic exam reported lower symptoms of post-traumatic stress (PSS-SR; CR = -3.45, 90% CI for *B: -18.95 to -2.75, r* = *-0.28*) and depression (BDI; CR = -2.88, 90% CI for *B: -18.89 to -1.04, r* = *-0.24).* No significant differences were observed at 6-months post-assault for post-traumatic stress, but the video was still associated with lower symptoms of depression with a smaller effect size (CR = -1.54, 90% CI for *B: -14.40 to -3.61, r* = *-0.13).* However, for those *without* a history of prior sexual assault, small effects indicating higher symptoms of PTSD (CR = 1.32; 90% CI for *B*: -3.50 – 10.87; *r* = 0.11) and anxiety (BAI; CR = 1.71; 90% CI for B: -3.03 to 14.89, *r* = 0.15) were found at 6-weeks post-assault for those in the video condition versus standard care (forensic exam with rape crisis counsellor present). This difference was not maintained at 6 months and there was no effect on depression scores in this group. This result could indicate that the video provided a lens for participants to evaluate their experience, providing normalising information for those with a prior history of sexual assault and prescribing symptoms or raising anxiety for those without. Alternatively, increased vulnerability and symptom burden in those with a prior history may imply greater scope for improvement, with previous experiences making the information in the video more relatable. This study was at high risk of bias.

Miller et al (2015), found that, for women ⩾18 years attending hospital for a forensic medical exam within 72 hours of assault, viewing the psychoeducational component of the video *after* the forensic exam (vs. standard care as above) was associated with reduced anxiety (STAI) for all participants at both the 2-week (mean diff = 8.60, SE diff = 3.41, p < .05) and 2-month follow-ups (mean difference = 6.66, SE diff = 3.11, *p* < .05). In contrast to Resnick et al., (2007b), at the 2-week follow-up women *without* a previous rape history who saw the video evidenced significantly lower post-traumatic stress scores (PSS-SR) than those *with* a previous history of rape (mean difference = 12.61, *p* = .011), suggesting intervention timing may be important, perhaps connected to anxiety levels while waiting for the exam. The risk of bias in this study was unclear, it had a small sample size, high dropout rate and a more inclusive definition of prior sexual assault history. As the complexity of an individual’s trauma history may feasibly impact on intervention efficacy this may have impacted results.

#### Effectiveness of video interventions by outcome

##### Post-traumatic stress

One study with unclear risk of bias found no significant impact on trauma symptoms or distress. A second study at high risk of bias reported a reduction in symptoms at 6-weeks post assault for those without a prior assault history, but higher symptoms in those without this history, with no difference at 6-months.

##### Depression

One study at high risk of bias found that women with a prior sexual assault history viewing the video before the forensic exam was associated with less depression symptoms at 6 weeks and 6 months.

##### Anxiety

One study with unclear risk of bias found no impact of video intervention on anxiety at 2-week and 2-month. A second found that the video increased anxiety in those without a prior sexual assault history at 6-weeks post-assault. No effect for those with a prior sexual assault history.

##### Alcohol and substance misuse

One study with unclear risk of bias found that *Any video* was associated with reduced marijuana use for those using prior to the sexual assault across a 6-month period. A second higher quality study found that the video was associated with lower alcohol and marijuana use, dependent on past year binge drinking/marijuana use and interacting with prior sexual assault history. Neither study found any effect of the intervention on problematic alcohol or drug use.

#### Summary of evidence for video interventions

Taken together these studies provide weak evidence that a CBT based video intervention may be effective at addressing the impact of trauma or preventing problematic drug or alcohol use before it becomes established. However, it is not clear which groups may benefit and a more tailored approach may be needed for people with more complex histories or where there is an established pattern of substance misuse. The potential for iatrogenic effects observed by Resnick et al. (2007b) is concerning and it may be important to consider timing to ensure maximum benefit. This brief, standardised, low cost intervention delivered at point of access has potential, but further robust research is needed.

#### Effectiveness of individual CBT-based interventions

Six studies evaluated CBT-based early interventions. Three aimed to prevent later psychological problems, providing between 1 and 6hrs intervention time (Kilpatrick and Veronen, 1984; Rothbaum et al., 2012; Tarquinio et al., 2012). Three studies trialled intensive treatments, 5–14 hours therapy, to treat acute symptoms and facilitate recovery (Anderson and Frank, 1991; Echeburúa et al., 1996; Nixon et al., 2016). Individual studies are presented and their contribution to the evidence discussed.

Tarquinio et al. (2012) provided psychoeducation on the impact of sexual trauma and provided one session of Eye Movement Desensitization and Reprocessing (EMDR) to women 24-72 hours after first sexual assault referred to a French research centre. EMDR involved imaginal exposure to the traumatic memory, focusing on points of high emotional distress while attending to therapist directed lateral eye movements, until distress reduced. The aim is resolution and integration of all aspects of the traumatic experience (Shapiro, 2001). They found significant pre-post improvement on post-traumatic symptoms (IES; total Wilks’s λ score (3, 14) = .09, p < .001) and Subjective Units of Distress (SUDs; Wilks’s λ (3, 14) = 069, *p* < .001), but non-significant changes at 4-week or 6-month follow-up. Self-reported sexual desire (Wilks’s λ (3,14) = 0.12) and excitation (Wilks’s λ (3,14) =0.09) improved after four weeks (*p* < .001) then stabilised. The study was at high risk of performance and detection bias as it employed a single group design with outcomes assessed within the therapeutic intervention, therefore, it provides weak evidence of short-term effect.

Rothbaum et al. (2012) used a modified Prolonged Exposure (PE) treatment protocol with women presenting to a hospital emergency department within 72 hours post-assault compared to assessment only. The PE intervention included breathing relaxation, in-vivo exposure, attention to cognitions, self-care strategies and repeated exposure to the trauma narrative to allow for fear extinction within and between sessions. The aim is to facilitate emotional processing and habituation to trauma memories. Those receiving brief PE reported significantly less PTSD with a large effect size at 4 weeks (PSS-I, Cohen’s d = 0.7, p < .01) and 12 weeks (d = 0.52, *p* = .05) post-assault. This was a well conducted study with various measures in place to reduce the risk of bias, however, attrition rates and mean age were not reported for the small sub-sample of rape victims. Acute stress was higher in the intervention group, which may have allowed more scope for improvement (controlled for statistically).

Two CBT based studies evaluated the impact of interventions on depression and fear for women less than one-month post-assault attending rape crisis centres. Anderson and Frank (1991) examined CBT, Systematic Desensitisation (SD; using imaginal exposure with relaxation to de-condition fear responses), brief Psychoeducation (PEI) and Psychological Support (PS). They reported significant reductions with *all* interventions in *depression* and *fear*, over the 1-year follow-up (*p* < .0001), with no significant differences between groups, suggesting brief interventions were equivalent to intensive treatments. However, risk of bias was unclear due to limited reporting and there was no control for spontaneous remission.

In contrast, Kilpatrick and Veronen (1984) found no significant benefit of a Brief (cognitive) Behavioural Intervention Procedure (BBIP) for women attending a rape crisis centre 6–21 days post-assault. BBIP included imaginal exposure, psychoeducation and coping skills training, compared to assessment only or repeated assessment controls over a three-month period. Outcomes included fear, sexual dysfunction, depression and anxiety. Risk of bias was unclear as methodological details were not reported.

Two studies employed individual psychological interventions for recent sexual assault survivors who met the DSM-IV criteria for Acute Stress Disorder (ASD). Echeburúa et al. (1996) compared Cognitive Restrucuting and Coping Skills (CR/CS; includes psychoeducation, modifying unhelpful thinking, coping skills such as progressive muscle relaxation or distraction and instruction in graded exposure) with Progressive Muscle Relaxation Training (PR) only. They found some benefits of CR/CS over PR, with significantly less severe PTSD symptoms, reported at 12month follow-up (*t* = 2.30, *p* < .05) and a trend up to this point. This change appears to be linked to reductions in re-experiencing and avoidance as indicated by subscale scores on the PTSD measure. There were no other significant between group differences on measures of depression, anxiety, fears and inadaptation, or in numbers meeting diagnostic criteria for PTSD at any time point. While, the reduction in PTSD symptomatology is promising the study had a very small sample (n=20) and was at high risk of bias.

Nixon et al. (2016), compared Brief Cognitive Processing Therapy (B-CPT; cognitive restructuring and trauma processing) with active treatment as usual (eclectic practices delivered by community clinicians working in a crisis centre, not systematic CBT/exposure) and found that both groups demonstrated large and clinically significant reductions in PTSD symptoms (CAPS and PCL-S; large effect, d = 0.76–1.45), depressive symptoms (BDI; large effect, d = 0.42–0.92) and in post-traumatic cognitions (PTCI; large effect, *d* = 0.42–0.94) at each follow up. Smaller, but reasonably consistent, between group effect sizes typically favoured B-CPT (ES: 0.13–0.50 for PTS and 0.13–0.41 depression) over the 12month follow-up period. All 12 comparisons favoured B-CPT, but effect sizes were small and confidence intervals were large representing variability across the small sample. The study was of good methodological quality with appropriate allocation concealment, use of an active control, monitoring of treatment adherence, blinding of outcome assessors and use of an intention to treat analysis. However, the method of randomisation was not reported, and treatment intensity was greater in the TAU condition which could have favoured this condition. This study provides evidence that an evidence-based trauma focused therapy such as CPT or more traditional approaches to rape crisis counselling can be effective when delivered as an early intervention for ASD.

#### Effectiveness of individual CBT-based interventions by outcome

##### Post-traumatic stress symptoms

Four studies reported a decrease in the severity of post-traumatic symptoms at follow-ups. Two studies were at low risk of bias, however, the sub-sample of rape victims in one was small. Two studies were at high risk of bias.

##### Depression

Two of four studies assessing impact on depression symptoms found a positive impact in terms of reduced symptoms at follow-ups. One had an unclear risk, the other low risk of bias. The other two studies found no effect and were of high or unclear risk.

##### Anxiety

Two studies with high or unclear risk found no impact on anxiety symptoms.

##### Rape-related fear

One study with unclear risk of bias found a positive impact of all interventions on fear. Two additional studies found no impact and were of poor methodological quality.

##### Sexual function

One study found no effect and one found a positive impact over the first 4-weeks; however, this study lacked a control and was at high risk of bias.

#### Summary of evidence for individual CBT-based interventions

Together these studies provide tentative support for the use of early CBT-based interventions in reducing or preventing post-traumatic stress, with the strongest support found for multi-session treatments involving exposure and processing of the trauma such as B-CPT and modified PE. These interventions were used with women presenting with high levels of acute distress who are also at the greatest risk of developing PTSD (Steenkamp et al., 2012). However, the diverse approaches, small sample sizes, differing treatment intensity and outcome measures used make it difficult to draw strong conclusions about effectiveness. The evidence for the effectiveness of interventions on outcomes such as depression, fear, anxiety or sexual function was weak and inconclusive.

## Discussion

Sexual assault and rape are particularly traumatic events which carry a high toll of psychological distress in their immediate aftermath and, for many, lead to ongoing psychopathology and disruptions in psychosocial functioning. However, little evidence exists about which psychosocial interventions employed soon after a sexual assault work and for whom, a challenge for those working in a first response or rape crisis setting. This systematic review found evidence that psychosocial interventions informed by cognitive behavioural models, particularly those including trauma exposure, warrant further rigorous investigation, particularly in reducing post-traumatic symptom severity.

Ten quantitative empirical studies met the inclusion criteria and examined outcomes including post-traumatic stress, depression, anxiety, fear, sexual function and substance use. Overall, the current evidence base supporting the effectiveness of psychosocial interventions for recent sexual assault survivors is limited and of poor scientific quality. Most studies were subject to a high risk of bias, resulting in poor internal and external validity, reflecting common limitations in their design. The lack of robust evidence to support study findings highlights the need for rigorous research in this area.

Evidence to support the use of an educational video intervention at the time of the forensic medical exam was mixed, with some evidence of beneficial effects on psychological functioning, dependent on prior assault history. Conflicting findings may be due to the different video components used, intervention timing, or due to factors related to previous sexual assault (e.g. existing symptoms of PTSD). The possibility that the full video intervention may be related to short-term iatrogenic effects on post-traumatic stress symptoms and anxiety in those without a previous rape history is concerning. There was some evidence that video interventions may help reduce substance misuse (marijuana and alcohol), particularly where problematic use has not yet become established. In summary, the evidence for the effectiveness of the video intervention(s) is inconclusive and, while potentially offering a low-cost, wide-reaching intervention, requires further examination, ideally with reference to some underlying theoretical basis.

There is tentative, but weak and inconclusive evidence to support the use of early CBT interventions. Four studies provided support for the use of CBT based interventions in reducing or preventing PTSD symptoms (Echeburúa et al., 1996; Nixon et al., 2012; Rothbaum et al., 2012; Tarquinio et al., 2012). The strongest support was found for treatments involving exposure and processing of the trauma such as brief-CPT or PE (Nixon et al., 2012; Rothbaum et al., 2012). These studies collectively suggest that trauma focused CBT interventions may accelerate recovery from Acute Stress Disorder and, with no reported adverse effects, this is promising.

The evidence supporting the use of CBT-based interventions in reducing depression, anxiety or rape related fears is lacking in the reviewed studies, with only one study showing any effect, albeit a good quality study with an active TAU (Nixon et al., 2016). Interventions trialled may not adequately address low mood or other factors may be influencing this. Researchers have applied an ecological lens to sexual assault recovery (e.g. Campbell et al., 2009), emphasising the stigmatising and blaming social context in which sexual assault occurs (Edwards et al., 2011). Survivors tend to internalise stigma, leading to self-blame, shame and unwillingness to report/seek help (Kennedy and Prock, 2018) or increased distress (Breitenbecher, 2006). These factors may distinguish rape trauma and account for the higher incidence of depression and increased suicide risk (Connor and Davidson, 1997; Kimerling et al., 2002). Viewing the consequences of rape as a matter of psychopathology may de-contextualise women’s gendered experience of sexual violence and may restrict our understanding (Wasco, 2003).

### Methodological challenges in this field

The high risk of bias in included studies has been discussed and renders the findings inconclusive. Further work of a higher methodological quality, with larger sample sizes, inclusion of appropriate controls, reporting details to enable evaluation of evidence, improved delivery checks and use of core measures for ease of comparison would be required to move towards a stronger evidence base. While minimising bias in an applied crisis context is difficult to achieve, Nixon et al. (2016) and Rothbaum et al., (2012) provide examples of good practice. Future RCTs with treatment adherence and integrity checks might compare CBT-based interventions based on trauma exposure with a comparable control drawn from current clinical practice or at least a time and attention control. Post-traumatic stress, subjective units of distress, depression and anxiety would ideally be measured over at least three time points.

The review included a broad study population, although it was overwhelmingly female, sexual assault and rape survivors were from diverse backgrounds, with complex needs including previous trauma history and psychiatric co-morbidity. These factors may influence which interventions are most likely to be effective, and specialised interventions may be required depending on need (e.g. Courtois and Ford, 2009). There is a need for research including male participants, gender or sexual minorities (Dworkin et al., 2017).

### Strengths and limitations of this review

This review identifies an important gap in the literature in relation to early interventions following sexual assault to improve wellbeing outcomes. This topic is relevant and timely given the increasing media and societal attention given to the implications of sexual victimisation which may well lead to an increase in the numbers presenting to services who in turn need to work from the best evidence. However, it is important to bear in mind that most victim/survivors do not report their assault in the immediate aftermath (Lanthier et al., 2018) and disclosure is mostly to friends or family, not services. Therefore, those presenting within the included studies may not represent the majority of victim/survivors.

This review was reported following the PRISMA guidelines and the risk of bias was comprehensively assessed and reported both individually and across studies. However, the eligibility criteria were quite broad concerning intervention, comparison, outcomes and study design. This was done with the intention of capturing a wide range of studies that might reflect current practice in this field, alongside more traditional psychological intervention studies. As a result, the authors are confident that studies evaluating interventions such as case management or crisis hotlines would have been included if they were to be found in the literature although it is possible a wider search strategy may have identified additional studies. The small number of studies, their considerable methodological flaws and heterogeneity begs the question as to whether study results can be compared and summarized effectively. The studies found in this review were all from the USA, Europe or Australia. This may be due to the inclusion of English papers or simply due to a lack of research in alternative contexts, but this may limit the relevance of these findings to a westernised context.

## Conclusion

This systematic review found inconclusive evidence for the effectiveness of psychosocial interventions with people who have experienced a recent sexual assault and clearly documents a gap in the evidence base for this field. Studies lacked methodological rigour, with a dearth of adequately powered, controlled studies to establish the effectiveness of interventions for various population groups, over and above routine care, or no treatment. Study details were often lacking. This represents a challenge for researchers and clinicians but points to more standardised approaches carried out to higher scientific quality standards. Future work should take as a starting point trauma-focussed CBT based interventions, and evaluate for whom, and to what degree these interventions are effective.

## Appendix 1: Further methodological details

### Databases searched

Assia, British Nursing Database, Campbell Collaboration, Cinahl, Cochrane Collaboration, Directory of Open Access journals, Emerald, Ethos, HMIC, Internurse, Medline, NICE, OpenSIGLE, PILOTS, ProQuest Dissertations and Theses, PsychARTICLES, PsychINFO, SAGE Journals Online, ScienceDirect, Social Care Online, Social Policy and Practice, Social Services Abstracts, SocINDEX, Taylor and Francis, SCOPUS, Wiley Online Library and Zetoc.

### Journals hand searched

The Journal of Interpersonal Violence, Violence against Women, Psychology of Women Quarterly, Trauma, Violence and Abuse and the Journal of Traumatic Stress.

### List of Previous review articles searched

Campbell et al., 2011; Decker and Naugle, 2009; Dworkin and Schumacher, 2016; Elderton, Berry and Chan, 2017; Martin et al., 2007; Parcesepe et al., 2015; Regehr et al., 2013; Taylor and Harvey, 2009; Vickerman and Margolin, 2009

### Main search terms used

(Sex* Assault OR Rape OR Sex* Violence OR Non-consen* OR Forced sex* OR Coerced Sex* OR post-rape OR sexual victim*) AND (Psycho* OR Suppor* OR Group OR Self-help OR Advocacy OR Social OR Intervention OR Peer OR Prog* OR Cent* OR Team OR Helpline OR Liaison OR Worker OR online OR case-tracker OR Social OR Ed* OR Couns* OR Respon* OR Train* OR Hotline OR Servic* OR Aftercare OR Treat* OR Preven*)

### Table of search terms and fields searched for each database searched at each timepoint

*nb HMIC and SocIndex were not available to the authors at the update searched.

**Table:**

Database Searched	Field Searched	Search Terms
ASSIA	Abstract or Title searched separately	Full search terms as above
British Nursing Index	Abstract or Title searched separately	Full Search terms as above
Campbell Collaboration	All	‘Sexual Assault’ only
CINAHL Plus	Title	Full search terms as above
Cochrane Collaboration	Title, abstract, keyword	Full search terms as above
Directory of open access journals	Searched Abstract English only Sexual assault string only	Sex* Assault OR Rape OR Sex* Violence OR Non-consen* OR Forced sex* OR Coerced Sex* OR post-rape OR sexual victim*
Emerald	Title Or Abstract	Sexual assault terms entered in separate lines of advanced search
EThOS	Abstract	Sexual assault OR rape OR sexual violence AND psychological OR psychosocial OR support **Then swap in intervention terms AND:** [group OR self help OR advocacy] [social OR intervention OR peer] [programme OR centre OR team] [helpline OR liaison OR worker] [online OR case tracker OR social] [education OR counselling OR response] [training OR hotline OR service] [aftercare OR treatment OR prevention] **THEN:** Non consensual sex OR forced sex OR coerced sex OR post rape OR sexual victimization OR sexual victimisation
HMIC	All fields	Full search terms
Internurse	All fields	Full search terms
Medline via OVID	Title only	Full search terms as above
NICE	Searched primary evidence only	Sexual assault search string only
OpenSIGLE (OpenGrey)	All search	Full search terms
PILOTS	Title	Full search terms
ProQuest Dissertations and Theses	Title	Full search terms
Psych ARTICLES (APA)	Title	Full search terms
PsychINFO	Title	Full search terms
SAGE Journals Online	Title	**Title**: (Sex* Assault) OR Rape OR (Sex* Violence) OR (Non-consen*) OR (Forced sex*) OR (Coerced Sex*) OR (post-rape) OR (sexual victim*) **AND** **Title**: Psycho* OR Suppor* OR Group OR (Self-help) OR Advocacy OR Social OR Intervention OR Peer OR Prog* OR Cent* OR Team OR Helpline OR Liaison OR Worker OR online OR (case-tracker) OR Social OR Ed* OR Couns* OR Respon* OR Train* OR Hotline OR Servic* OR Aftercare OR Treat* OR Preven*
ScienceDirect	Title, Abstract, Keyword	(“Sexual Assault” OR Rape OR “Sexual Violence” OR “Non consensual”) AND. . . (“Forced sex” OR “Coerced Sex” OR “post rape” OR “sexual victim”) AND. . . . **Coupled with these sequentially** [. . . . . . .AND (Psychological OR Support OR Group OR “Self help” OR Advocacy)] [. . .AND (Social OR Intervention OR Peer OR Programme OR Centre)]
		[. . .AND (Team OR Helpline OR Liaison OR Worker OR online)] [. . .AND (case-tracker OR Social OR Education OR Counselling OR Response)] [. . .AND (Training OR Hotline OR Service OR Aftercare OR Treatment)] [. . .AND (Prevention)]
Social Care Online	All	Full search terms
Social Policy and Practice	Title	Full search terms
**Social Services Abstracts**	Title	Full search terms
**SocINDEX**	Title	Full search terms
**Taylor & Francis**	Title	(“Sex* Assault” OR Rape OR “Sex* Violence” OR “Non consen*” OR “Forced sex*” OR “Coerced Sex*” OR “post rape” OR “sexual victim*”) AND (Psycho* OR Suppor* OR Group OR “Self help” OR Advocacy OR Social OR Intervention OR Peer OR Prog* OR Cent* OR Team OR Helpline OR Liaison OR Worker OR online OR case-tracker OR Social OR Ed* OR Couns* OR Respon* OR Train* OR Hotline OR Servic* OR Aftercare OR Treat* OR Preven*)
**SCOPUS**	Title	TITLE ( sex* AND assault OR rape OR sex* AND violence OR non-consen* OR forced AND sex* OR coerced AND sex* OR post-rape OR sexual AND victim* ) AND TITLE ( psycho* OR suppor* OR group OR self-help OR advocacy OR social OR intervention OR peer OR prog* OR cent* OR team OR helpline OR liaison OR worker OR online OR case-tracker OR social OR ed* OR couns* OR respon* OR train* OR hotline OR servic* OR aftercare OR treat* OR preven* )
**Wiley Online Library**	Title	Full search terms
**Zetoc**	Title	Sexual assault paired sequentially with intervention; psycho*; servi*; suppor*; advocacy; helpline; worker THEN rape paired with the same terms

### Data Extraction Form details

The data extraction form was based on the on the Cochrane RCT and non-RCT form (Available from https://dplp.cochrane.org/data-extraction-forms). Data was extracted about the study aim, population, setting, inclusion and exclusion criteria, recruitment, randomisation, withdrawals and exclusions, participant characteristics, baseline differences, intervention details, outcome measures (including psychometric properties), time points, assessor details, statistical methods, power calculations and the applicability of the study.
